# A survey of self-reported varicose veins with prior clinical diagnosis among adults residing in the United Arab Emirates: a cross-sectional study examining associated factors

**DOI:** 10.3389/fepid.2026.1916966

**Published:** 2026-09-11

**Authors:** Riya Inder Sevhani, Vivek Vasudevan, Diya Harish Vanjani, Prasanna AP, Aashna Anuraag Mewada, Dhyan Ashish Shah, Kashish Doulatram Mohandas Devnani, Shahnaz Mohammad Wazil, Anusha Sreejith

**Affiliations:** 1 College of Medicine, Gulf Medical University, Ajman, United Arab Emirates; 2 College of Medicine, Medical University of Lublin, Lublin, Poland; 3 Biomedical Science, College of Medicine, Gulf Medical University, Ajman, United Arab Emirates; 4 Department of Community Medicine, Gulf Medical University, Ajman, United Arab Emirates; 5 Thumbay Institute of Population Health, Gulf Medical University, Ajman, United Arab Emirates

**Keywords:** advancing age, body mass index, family history, prevalence, United Arab Emirates, varicose veins

## Abstract

**Background:**

Varicose veins are a common manifestation of chronic venous diseases. Despite their clinical and occupational relevance, epidemiological data from the United Arab Emirates remains limited. This study estimated the prevalence of self-reported clinically-diagnosed varicose veins and examined the associated factors among adults residing in the UAE.

**Methods:**

A cross-sectional survey was conducted across all seven emirates of the UAE between June 2025 and April 2026. A total of 1,043 eligible participants residing in the UAE were recruited through non-probability convenience sampling technique, and findings should be interpreted as preliminary and non-generalizable. The prevalence of varicose veins was estimated based on self-reported prior clinical diagnosis. Data was analyzed using SPSS (v29), associations with demographic, lifestyle, and medical factors were assessed using Pearson's chi-square tests and logistic regression models.

**Results:**

Among surveyed adults in the UAE, 15.05% self-reported a prior clinical diagnosis of varicose veins; this figure reflects the study sample only and should not be interpreted as a population-representative prevalence. Most participants correctly identified varicose veins. Advancing age, body mass index, prolonged standing, and positive family history of the disease were significantly associated with higher odds (*p* < .001).

**Conclusion:**

These findings provide preliminary, non-generalizable evidence on self-reported previously diagnosed varicose veins among the surveyed convenience sample of adults in the UAE. The condition represents a notable concern within the study population, and the results are hypothesis-generating, emphasizing the need for targeted early diagnosis among high-risk individuals, while also highlighting the need for clinically validated, probability-based population studies in the UAE.

## 1 Introduction

Varicose veins are visible, enlarged, twisted, and painful superficial veins that commonly affect the lower limbs, arising due to venous valve insufficiency and weak vein walls, causing blood to pool and vessel walls to distend abnormally (1). They reflect underlying chronic venous diseases, which are conditions arising due to impaired venous return in lower extremities, and are associated with significant morbidity, diminishing overall quality of life (2). The pathophysiology of varicose veins is complex and involves mechanisms that causes venous blood reflux and stasis, leading to progressive structural changes in the vein wall (3–5). The condition follows a chronic course and may lead to complications like venous ulcers, deep vein thrombosis, and chronic venous insufficiency if left untreated (6, 7). However, varicose veins are commonly perceived as a cosmetic concern, and early clinical symptoms like leg pain, heaviness, skin discoloration, itching, and muscle cramps are often normalized (8, 9).

Varicose veins affect approximately one-third of adults globally, with estimates suggesting that global prevalence lies between 10% and 30%, though some populations report even higher rates depending on their methodology and study criteria (10). This represents a significant public health burden. Epidemiological literature has shown that biological factors like genetic predisposition, advancing age, and female sex increase the risk and progression of the disease (11). Women are at a higher risk of developing varicose veins as compared to men, although high prevalence rates have been reported among men as well (12, 13). Lifestyle and occupational exposures, particularly involving prolonged standing, physical inactivity, and obesity are modifiable risk factors that are consistently associated with a higher prevalence of the condition (14, 15).

In the Middle Eastern region, several characteristics make resident populations more vulnerable to venous diseases. Multiple studies conducted in Saudi Arabia have reported epidemiological data on the prevalence and risk factors of varicose veins, suggesting prolonged standing and body mass index (BMI) are significant predictors (2, 3, 8). The diverse population in the UAE, predominantly composed of expatriates who engage in either physically demanding occupations or sedentary lifestyles, remains largely unexplored in the context of varicose veins (6, 11). Furthermore, extreme environmental temperatures and rising obesity rates in the UAE may exacerbate venous dysfunction through peripheral vasodilation and venous stasis (14). These regional characteristics underscore the importance for dedicated epidemiological studies in the Middle Eastern region, particularly in the United Arab Emirates.

Despite this relevance, no community-based epidemiological study has yet reported the prevalence of varicose veins in the UAE. Existing evidence is largely confined to small hospital-based studies within neighboring countries, limiting the generalizability of their findings to the UAE's distinct demographic and environmental context. Due to these reasons, public health interventions addressing preventive strategies and targeting vulnerable populations are limited. These cumulative gaps highlight the significance of conducting this research. Therefore, the present study aims primarily to estimate the self-reported prevalence of varicose veins based on prior medical diagnosis among adults residing in the UAE and, secondarily, to identify the associated demographic, lifestyle, and medical factors.

## 2 Methods

### 2.1 Study design, setting, and sampling

A cross-sectional study design was chosen, and the study was conducted across all seven emirates of the United Arab Emirates from June 2025 till April 2026. The sample size was calculated using the formula for single proportion estimation, based on an expected prevalence of 0.33 as reported by Antani MR et al. (16), with 10% relative precision, yielding a minimum required sample size of 1,012 participants. A further adjustment of a potential non-response rate of 10% was then applied, a standard adjustment for online/community-based surveys where incomplete or ineligible responses are common, yielding a target sample size of 1,120 participants. Recruitment was continued until this target was approached; however, responses that did not meet the predetermined eligibility criteria were excluded, resulting in a final eligible sample of 1,043 participants. This achieved sample size closely approximates the originally calculated target and is considered adequate to support the primary self-reported prevalence estimate.

### 2.2 Participant recruitment and eligibility criteria

**Figure 1:**
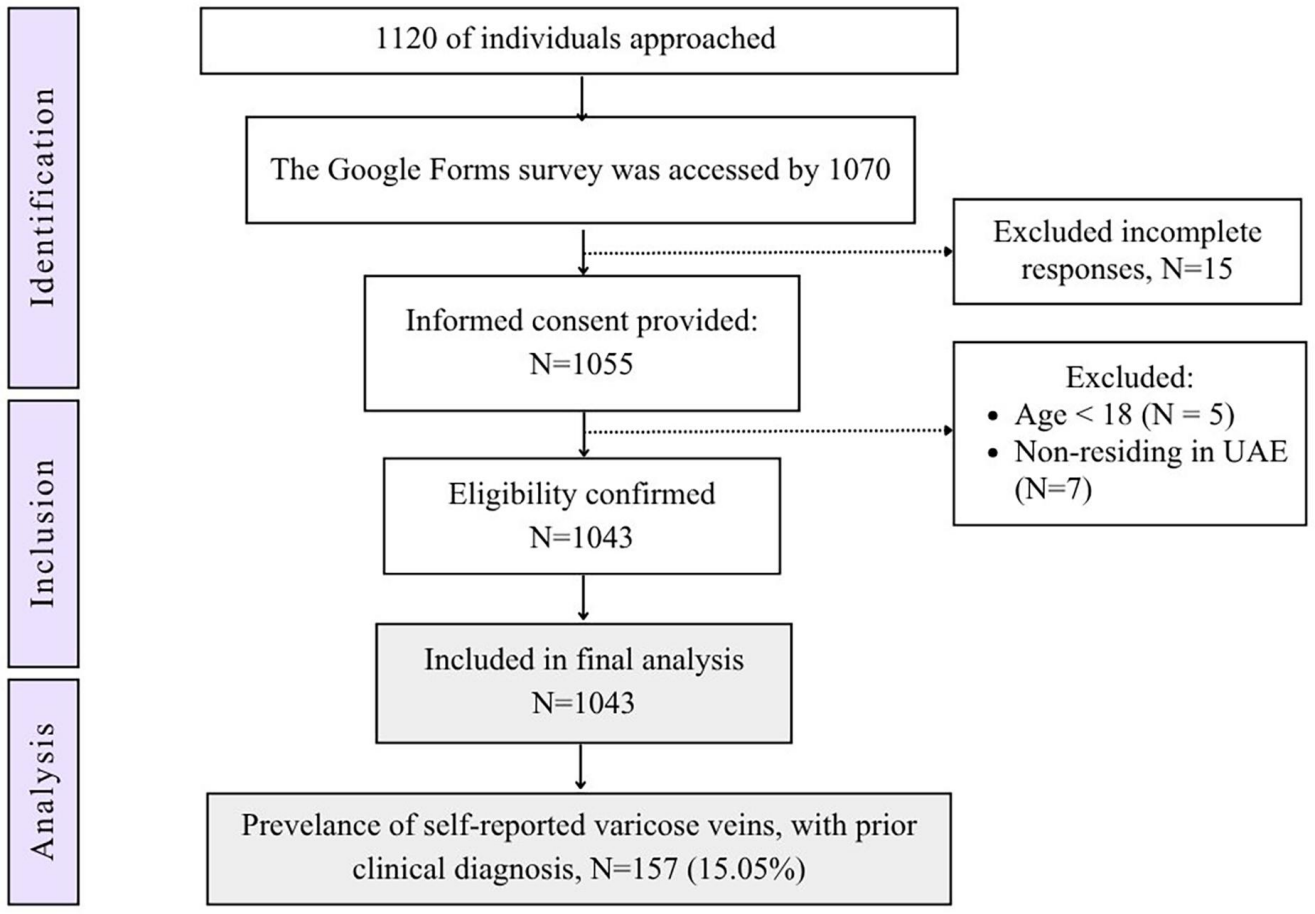
STROBE flow diagram of participant selection and inclusion.

A non-probability convenience sampling technique was used due to its practicality and feasibility for rapid data collection. Participants were recruited through online dissemination of the survey via multiple online platforms and social media channels, including WhatsApp, Instagram, community health groups, and university networks, and multiple community settings, including public leisure venues (shopping malls and cafes), were approached to enhance diversity. This approach aimed to improve the heterogeneity of the study sample. All seven emirates were represented in the final sample size, maximizing geographic coverage. Participant selection and inclusion criteria have been illustrated in Figure 1.

### 2.3 Variables assessed and measurements

Data was collected using a newly developed self-administered questionnaire via Google Forms. Three experts of vascular surgery and community medicine reviewed the questionnaire to ensure content validity and necessary modifications were made following their feedback. While the questionnaire underwent content validation by subject-matter experts, no formal psychometric validation was performed; this is acknowledged as a study limitation. A pilot study was conducted among 10 individuals to evaluate clarity and feasibility. All participants were shown standardized clinical images of varicose veins in lower limbs, which were reviewed and validated by experts to confirm accuracy and appropriate clinical representation.

The prevalence of varicose veins was estimated through self-reported prior clinical diagnosis of varicose veins. Sociodemographic (age, gender, BMI, residing emirate, nationality, education level, and employment status), lifestyle (exercise, smoking, alcohol, caffeine consumption, hydration, and average time spent standing and sitting per day) and medical factors (history of chronic conditions, micronutrient deficiencies, constipation, and family history of varicose veins) were self-reported by all participants. Visual awareness was operationally based on correct identification of the standardized clinical image representing varicose veins, and responses were categorized accordingly.

Lifestyle variables were operationally defined as follows: Exercise was categorized as regular engagement in structured physical activity of at least 150 min per week (yes/no); smoking and alcohol consumption were each categorized as current use (yes/no); caffeine consumption was categorized as low/none (≤100 mg/day) versus moderate-to-high (100–400 mg/day or more), estimated from self-reported number and type of caffeinated beverages consumed daily; hydration status was categorized by self-reported average daily water intake into three bands (less than 2 liters, 2–3 liters, and 3 liters or more); and average daily time spent standing and sitting were each self-reported using four categorical bands (less than 2 h, 2–4 h, 4–6 h, and more than 6 h).

### 2.4 Statistical methods

The dataset was analyzed using SPSS software version 29. Sociodemographic characteristics were summarized using descriptive statistics. Associations between categorical variables and prevalence were assessed by Pearson's chi-square tests, with statistical significance set at *p* < 0.05. Significant variables were further included in bivariate and multivariate logistic regression models. Crude and adjusted odds ratios (CORs and AORs) were reported with 95% confidence intervals (CIs).

### 2.5 Ethical considerations

Ethical approval was obtained from the Institutional Review Board of Gulf Medical University (Ref. no. IRB-COM-STD-182-June-2025). Informed consent was obtained, and participant anonymity and confidentiality were ensured. The study was conducted in accordance with the Declaration of Helsinki.

## 3 Results

### 3.1 Sociodemographic characteristics

The study included 1,043 participants from the UAE, as summarized in Table 1. Most of the participants (45.3%) were aged between 30 and 45 years, while females were slightly more than half (53.5%). A normal body mass index (BMI) was observed in 43% of participants. Among the diverse nationalities in the UAE, there was a predominance of the Southeast Asia region (68.5%).

**Table 1:** Sociodemographic characteristics (*N* = 1,043).

Variable	Group	Frequency	Percent (%)
Age	less than 30 years	289	27.7
	between 30 and 45 years	473	45.3
	above 45 years	281	27
Gender	Male	485	46.5
	Female	558	53.5
BMI	Normal weight	448	43
	Overweight	442	42.3
	Obese	153	14.7
Emirate of residence	Abu Dhabi	66	6.3
	Dubai	513	49.2
	Sharjah	255	24.4
	Ajman	169	16.2
	Umm Al Quwain	12	1.2
	Ras Al Khaimah	17	1.6
	Fujairah	11	1.1
Nationality	EMR – Eastern Mediterranean Region	176	16.9
	SEAR – South-East Asia Region	714	68.5
	WPR - Western Pacific Region	87	8.3
	Others	66	6.3
Education level	Primary school	37	3.5
	Secondary/high school	221	21.2
	Bachelor's	550	52.7
	Master's	210	20.1
	PHD	25	2.4
Employment status	No	259	24.8
	Yes	784	75.2

This table presents the descriptive statistics of the sociodemographic variables of the study population. BMI, Body Mass Index; Others = African Region (Nigeria, Kenya, Ethiopia, Sudan); European Region (UK, Germany, France); and Region of the Americas (USA, Canada, Brazil).

### 3.2 Self-reported prevalence of varicose veins

**Figure 2:**
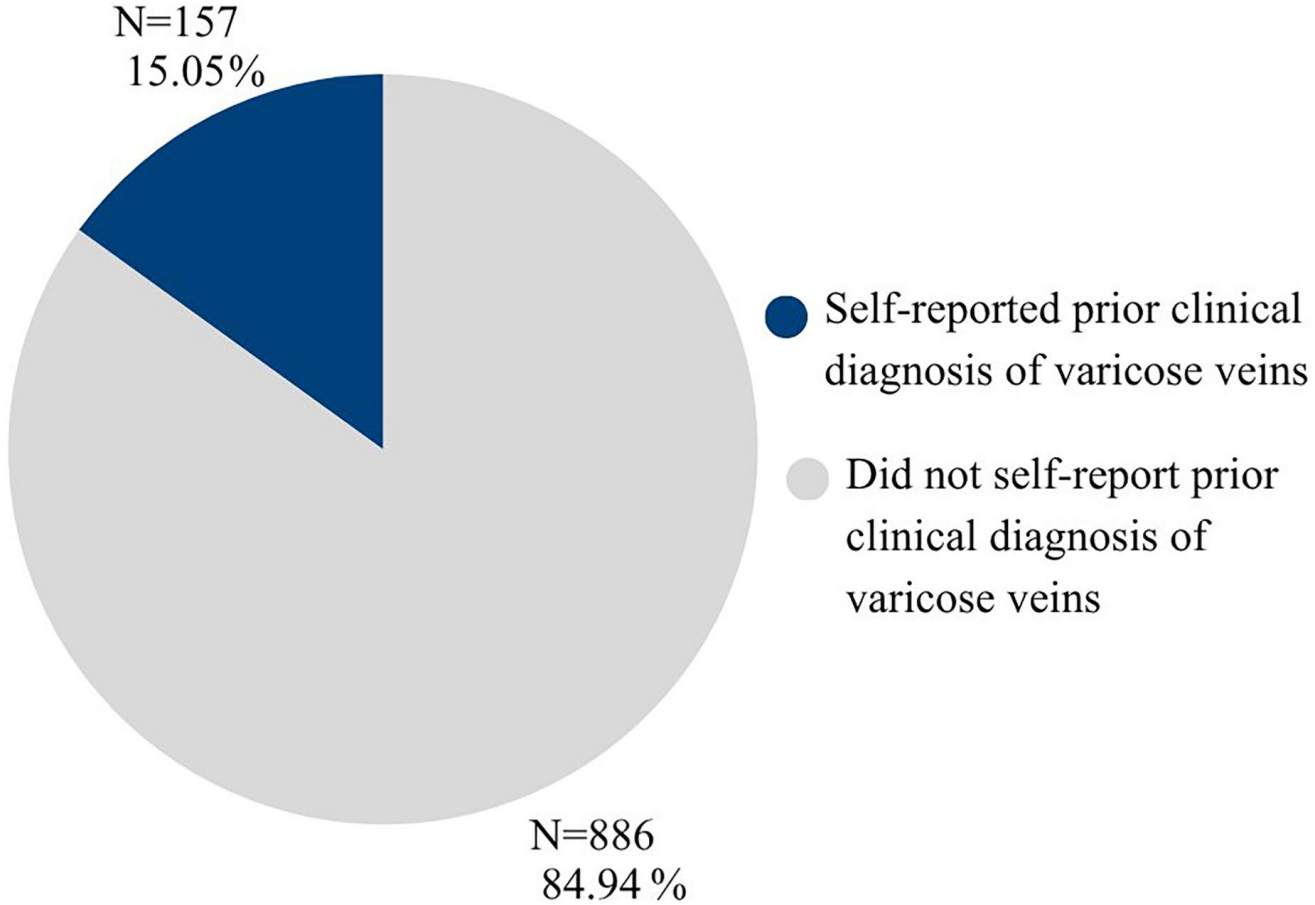
Prevalence of self-reported varicose veins with prior clinical diagnosis among adults residing in the UAE (*N* = 1,043).

The prevalence of self-reported varicose veins among surveyed adults in the United Arab Emirates was determined to be 15.05%. As illustrated in Figure 2, out of 1,043 participants, 157 had a prior medical diagnosis of varicose veins.

### 3.3 Visual awareness of varicose veins

**Figure 3:**
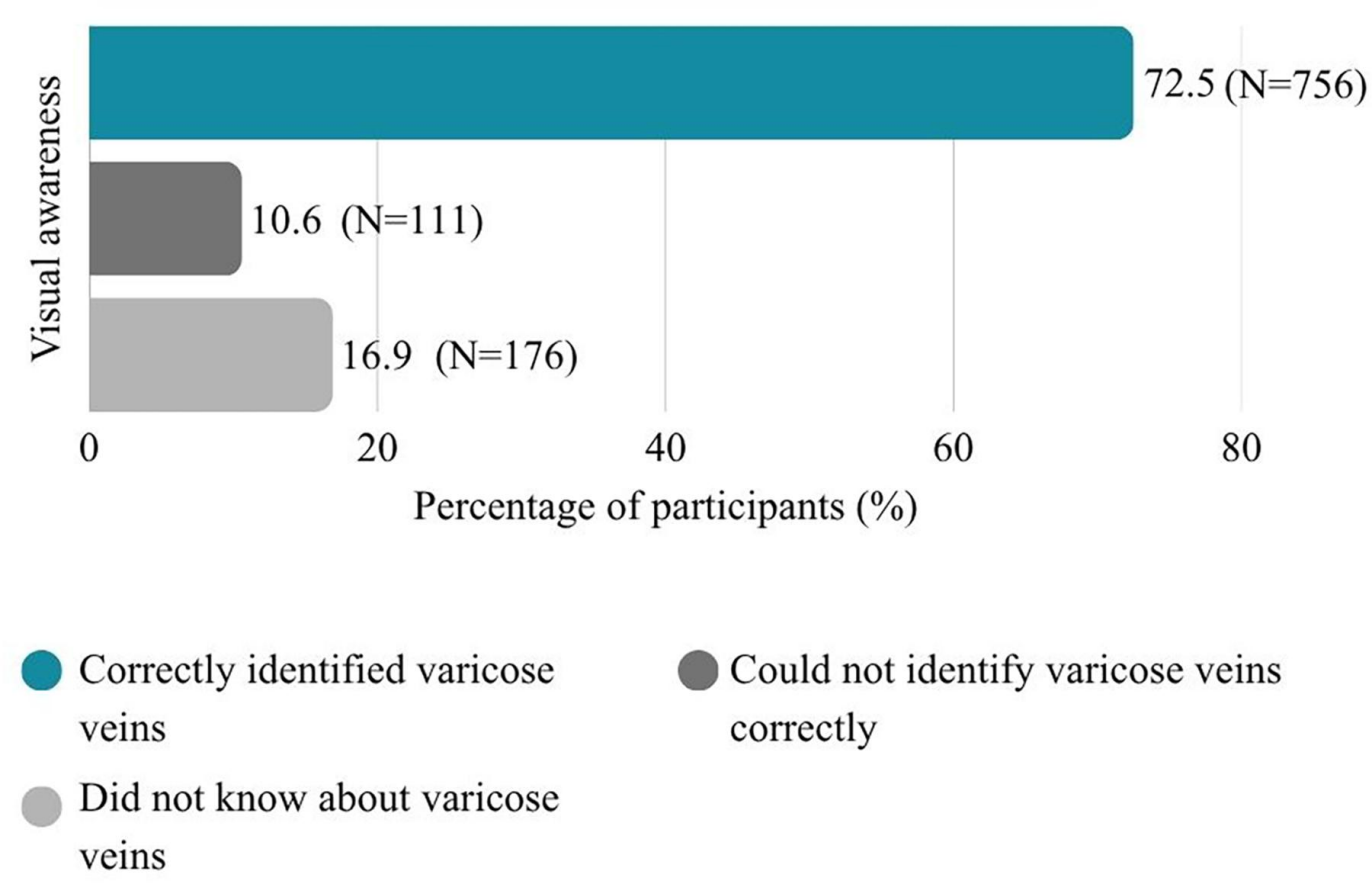
Visual awareness of varicose veins among study participants (*N* = 1,043).

Visual awareness was assessed using standardized clinical images, and 72.5% of individuals correctly identified varicose veins (Figure 3).

### 3.4 Bivariate logistic regression analyses showing the association of sociodemographic, lifestyle, and medical factors with the prevalence of varicose veins

Pearson's chi-square tests are provided in the supplementary material. Supplementary Table S1 shows the significant associations between sociodemographic characteristics like age, gender, nationality, and BMI with the prevalence of self-reported varicose veins with prior clinical diagnosis (*p* < .001). these associations were included in logistic regression analysis, as reported in Table 2, individuals aged between 30 and 45 years (OR=4.28, 95% CI: 2.28–8.03) and those above 45 years (OR=7.8, 95% CI: 4.12–14.76) had comparatively higher odds of developing the condition. Additionally, obese individuals also showed a similar association (OR=5.55, 95% CI: 3.43–8.97).

**Table 2:** Logistic regression analysis of significant sociodemographic variables (*N* = 1,043).

Sociodemographic variables		Crude Odds Ratio (OR)	Confidence Interval (CI) (95%)		*p*-value
			Lower	Upper	
Age	less than 30 years	1			
	between 30 and 45 years	4.281	2.283	8.030	<.001
	above 45 years	7.804	4.126	14.764	<.001
Gender	Male	1			
	Female	1.588	1.119	2.253	.010
Nationality	Others	1			
	EMR – Eastern Mediterranean Region	1.383	.724	2.644	.326
	SEAR—South-East Asia Region	.383	.208	.705	.002
	WPR - Western Pacific Region	.361	.148	.878	.025
BMI	Normal weight	1			
	Overweight	2.190	1.432	3.350	<.001
	Obese	5.556	3.439	8.976	<.001

BMI, Body Mass Index. Bivariate logistic regression was performed to identify significant predictors among sociodemographic factors associated with the prevalence of varicose veins. The reference category was assigned an OR of 1. A 95% confidence interval excluding 1 was considered indicative of statistical significance. *p*-values indicate statistical significance (*p* < 0.05).

Lifestyle factors like exercise, hydration habits, average time spent standing and medical factors like chronic diseases, nutrient deficiencies, and family history of varicose veins (*p* < .001) were significantly associated (Supplementary Table S2–S3) and further included in the logistic regression analysis. In Table 3, participants who had inadequate hydration (less than 2 liters) had higher odds (OR=2.44, 95% CI: 1.68–3.53). Prolonged standing (more than 6 h) also posed a significant risk (OR=3.64, 95% CI: 2.21–6.0). Multiple chronic diseases and low magnesium levels were significant as well. Genetic predisposition with a positive family history of varicose veins exhibited substantially higher odds (OR=14.16, 95% CI: 8.93–22.45).

**Table 3:** Logistic regression analysis of significant lifestyle and medical variables (*N* = 1,043).

Lifestyle Factors		Crude Odds Ratio (OR)	Confidence Interval (CI) (95%)		*p*-value
			Lower	Upper	
Exercise	Yes	1			
	No	1.748	1.224	2.496	.002
Hydration status	More than or 3 liters	1			
	Less than 2 liters	2.444	1.688	3.539	<.001
Caffeine consumption	Low or No (</=100 mg)	1			
	Moderate to high (100 mg to 400 mg or more)	2.039	1.377	3.019	<.001
Hours spent sitting daily	More than 6 h	1			
	Less than 2 h	2.131	1.195	3.801	.010
	2–4 h	1.496	.954	2.347	.080
	4–6 h	.763	.480	1.211	.251
Hours spent standing daily	Less than 2 h	1			
	2–4 h	.542	.293	1.002	.051
	4–6 h	1.235	.719	2.123	.444
	More than 6 h	3.648	2.217	6.003	<.001
*Medical Factors*					
Diabetes	No	1			
	Yes	3.141	2.084	4.734	<.001
Hypertension	No	1			
	Yes	2.997	2.081	4.316	<.001
High cholesterol	No	1			
	Yes	2.327	1.584	3.417	<.001
Arthritis	No	1			
	Yes	4.977	2.833	8.742	<.001
Hormonal diseases (Thyroid, PCOS)	No	1			
	Yes	2.537	1.651	3.899	<.001
Low Magnesium	No	1			
	Yes	3.418	2.157	5.417	<.001
Constipation	No	1			
	Yes	2.699	1.887	3.861	<.001
Family history of varicose veins	No	1			
	yes	14.160	8.932	22.450	<.001
	I do not know	2.133	1.203	3.779	.009

PCOS, polycystic ovarian syndrome. Bivariate logistic regression was performed to identify significant predictors among lifestyle and medical factors associated with the prevalence of varicose veins. The reference category was assigned an OR of 1; a 95% confidence interval excluding 1 was considered indicative of statistical significance. *p*-values indicate statistical significance (*p* < 0.05).

### 3.5 Multivariate logistic regression of significant sociodemographic, lifestyle, and medical factors with prevalence of varicose veins

As reported in Table 4, sociodemographic characteristics like age and BMI retained their statistical significance. Participants aged above 45 years had progressively higher odds of developing varicose veins (AOR=5.81, 95% CI: 2.56–13.18). Additionally, obese individuals were at higher risk. Standing for more than 6 h daily and a positive family history contributed to significantly higher odds as well.

**Table 4:** Multivariate logistic regression analysis (*N* = 1,043).

Sociodemographic factors		AORs	Confidence Interval (CI) (95%)		*p*-value
			Lower	Upper	
Age	less than 30 years	1			
	between 30 and 45 years	2.481	1.186	5.190	.016
	above 45 years	5.814	2.564	13.183	<.001
Gender	Male	1			
	Female	1.212	.705	2.086	.487
Nationality	Others	1			
	EMR – Eastern Mediterranean Region	5.336	1.958	14.540	.001
	SEAR—South-East Asia Region	.784	.317	1.937	.598
	WPR - Western Pacific Region	.965	.275	3.387	.955
BMI	Normal weight	1			
	Overweight	1.796	1.026	3.145	.040
	Obese	3.388	1.691	6.788	<.001
*Lifestyle factors*					
Exercise	No	1			
	Yes	1.129	.656	1.944	.661
Hydration	More than or 3 liters				
	Less than 2 liters	1.901	1.151	3.138	.012
Caffeine consumption	Low or No (</=100 mg)	1			
	Moderate to high (100 mg to 400 mg or more)	1.854	1.086	3.166	.024
Hours spent sitting daily	More than 6 h	1			
	4–6 h	.798	.398	1.603	.526
	2–4 h	1.117	.524	2.381	.775
	Less than 2 h	2.304	.893	5.948	.084
Hours spent standing daily	Less than 2 h	1			
	2–4 h	1.129	.492	2.593	.774
	4–6 h	2.921	1.311	6.508	.009
	More than 6 h	6.623	2.841	15.441	<.001
*Medical factors*					
Diabetes	No				
	Yes	2.226	1.151	4.305	.017
Hypertension	No				
	Yes	1.411	.777	2.563	.258
High cholesterol	No				
	Yes	.572	.297	1.101	.094
Arthritis	No				
	Yes	1.426	.611	3.328	.411
Hormonal diseases (Thyroid, PCOS)	No				
	Yes	1.638	.858	3.128	.135
Low Magnesium	No				
	Yes	3.413	1.716	6.788	<.001
Constipation	No				
	Yes	1.218	.712	2.084	.472
Family history of varicose veins	No				
	yes	16.290	9.023	29.409	<.001
	I do not know	2.274	1.179	4.389	.014

BMI, Body Mass Index; AOR, Adjusted odds ratio; PCOS, polycystic ovarian syndrome;.

Multivariate logistic regression was performed to identify independent predictors among the significant sociodemographic, lifestyle, and medical factors associated with the prevalence of varicose veins. The reference category was assigned an AOR of 1; a 95% confidence interval excluding 1 was considered indicative of statistical significance. *p*-values indicate statistical significance (*p* < 0.05).

## 4 Discussion

### 4.1 Prevalence and awareness

This study provides preliminary evidence on self-reported clinically-diagnosed varicose veins among a convenience sample of adults residing in the United Arab Emirates. The observed prevalence estimate of 15.05% falls within the lower range of internationally reported prevalence figures (17) and is comparable to various regional studies in the Middle East (18–20). However, an explicit comparison was not possible due to the absence of epidemiological data in the UAE. This study addresses a critical gap in literature and presents essential insights from within the country; however, as our outcome was based on self-reported clinical diagnosis collected via non-probability sampling, the findings should be interpreted as preliminary evidence rather than a population prevalence estimate.

The observed awareness was 72.5%, suggesting a substantial proportion of participants were able to visually identify the condition. A notable strength of our study is assessing awareness by utilizing standardized images. Recent studies have proven that visual and image-based health communication significantly enhances disease recognition, specifically in visually identifiable diseases like varicose veins (21, 22). However, this reflects general awareness rather than clinical understanding of the disease and does not ensure that affected individuals will seek medical evaluation if needed. This awareness-to-action gap may reflect structural barriers affecting the UAE's expatriate majority, including healthcare costs, occupational time constraints, and concerns regarding medical consultations, consistent with broader population health frameworks emphasizing the role of social capital, trust, and healthcare system responsiveness in shaping health-seeking behavior and disease detection across diverse populations (23).

### 4.2 Sociodemographic significance

Our results state that advanced age, greater than 45 years, increases the odds of disease development by 5 times. This association can be clinically explained by age-dependent vascular changes like progressive loss of endothelial cells, inflammation, and scarring of the weakened venous wall, which manifest as tortuous and dilated visible veins. Advancing age is also linked to thickening and stiffening of venous valves, leading to reflux and blood stasis in veins (24). However, older adults also have longer exposure time to diagnosis, which may partly inflate the observed association. Our findings are consistent with recent cross-sectional studies in Saudi Arabia and Egypt that concluded a significant age-related increase in incidence of the disease (19, 25). Our observations aligned with a German study conducted across a diverse population as well (26). For contrast in perspective, our findings were not consistent with a study in Saudi Arabia conducted among surgeons (27); however, these differences can be attributed to different occupational settings. Participants from the EMR nationality showed higher odds, although the underlying biological mechanism remains unclear, this association suggests EMR nationals may be more likely to receive clinical diagnoses relative to other nationalities, reflecting different healthcare utilization and awareness patterns across the study population (28).

BMI was significantly associated with varicose veins; obese individuals had nearly 3 times higher odds of acquiring the condition. These findings align with established pathological pathways linking adipose tissue deposition to chronic venous disease by elevating intra-abdominal pressure and venous pressure in lower limbs, causing symptoms of varicose veins. Furthermore, increasing BMI has a significant correlation with disease progression (29, 30). This is particularly relevant to the UAE, where nearly 58% of adults are overweight or obese, representing a substantial at-risk population (31). This association is consistent with regional evidence. A cross-sectional study in Saudi Arabia found that obesity was reported by 71% of those diagnosed with varicose veins (32). While differences in population demographics and methodology limit direct comparability of their estimates to the UAE setting, the consistent identification of obesity as a significant predictor reinforces the importance of weight management as part of venous disease prevention strategies.

### 4.3 Lifestyle and medical associations

Prolonged standing (more than 6 h daily) increased the risk by nearly 7 times (AOR=6.62). Biological mechanisms suggest that prolonged standing elevates venous hydrostatic pressure in the lower limbs, resulting in stress on the venous valves. Over time, this may contribute to valvular incompetence, venous reflux, and blood stasis (34, 35). A cross-sectional study in Iraq reported similar findings (36). Furthermore, recent systematic reviews and meta-analyses investigating the global epidemiology of varicose veins have consistently identified prolonged standing as an important modifiable risk factor (37–39). However, in contrast to our results, a cross-sectional study conducted by Soeder J et al. did not observe a statistically significant association between objectively measured occupational standing and varicose veins, although continuous prolonged standing was significantly associated with pathological venous reflux, suggesting that continuous standing without heterogeneous movement may lead to venous insufficiency. Notably, the authors acknowledged limitations like smaller sample size and occupational heterogeneity (40). These findings highlight the complex association between prolonged standing and venous disease and supports occupational-health interventions, including movement breaks, ergonomic redesign, and education among workers, the effectiveness of which depends substantially on healthcare system factors, including organizational commitment to worker health, availability of preventive services, and the extent to which providers are empowered to deliver occupational health guidance (41).

Low hydration status (less than 2 liters of water per day) was associated with higher odds. This finding is consistent with a regional case-control study conducted in Egypt, which reported similar findings (14). Given the UAE's hot climate and increased risk of dehydration, adequate hydration may represent a potentially modifiable factor that can be included in lifestyle-based prevention strategies for venous disorders (33). However, this association should be interpreted with caution, as water intake was self-reported and may not accurately represent physiological hydration status; the finding is therefore exploratory and hypothesis-generating, and further studies are needed to elucidate the underlying pathophysiological mechanisms linking hydration status with the condition. Lower magnesium levels were similarly associated, although they were self-reported; this finding is linked with the condition's coherent biological basis, as it exacerbates inflammation and destabilizes the extracellular matrix, resulting in vulnerability to venous wall injury. A clinical study directly examining this association found that low serum magnesium levels may pose a potential risk for the development of chronic venous insufficiency (42). However, this finding underscores the need for further evaluation through biomedical validation. The lack of independent associations for hypertension, high cholesterol, arthritis, hormonal disease, and constipation suggests that these factors may not be strong predictors of the disease and are influenced by confounding from demographic, lifestyle, or medical variables, resulting in attenuation of their effects after adjustment.

A positive family history was the most robust independent predictor (AOR=16.29). The genetic aspects of this disease have only been recently explored. Genome-wide association studies (GWAS) in chronic venous diseases have identified multiple susceptibility loci and genes with plausible biological significance in extracellular matrix regeneration, immune signaling, vascular development, and smooth muscle function. These genetic associations strongly support the widely observed trend of hereditary factors related to the development of varicose veins (43, 44). Several regional and global studies support these findings. In three cross-sectional studies carried out among surgeons, healthcare providers, and teachers of both sexes across Saudi Arabia and Jordan, a positive family history was reported as a significant risk factor for developing varicose veins (39, 45, 46). Furthermore, global systematic reviews and meta-analyses have consistently highlighted similar findings, emphasizing the epidemiological significance of genetic predisposition on the disease pathology and highlighting the importance of familial risk stratification in clinical and public health settings. However, family history was self-reported and may also reflect greater awareness and diagnostic vigilance within affected families.

### 4.4 Limitations

This study has limitations that should be considered while interpreting its findings. A cross-sectional design precludes establishing causal relationships between identified factors and the self-reported clinically diagnosed varicose veins. Further studies with suitable methodology and clinical validation are required to investigate this association. The use of non-probability sampling may have introduced a potential selection bias, limiting the generalizability of the findings to the wider UAE population. Certain demographics may have been over or underrepresented, potentially influencing the prevalence estimates. In particular, South-East Asian nationals comprised 68.5% of the study sample, which exceeds government-reported estimates of South Asian representation in the UAE population (approximately 50%–60%), while participants from the Eastern Mediterranean and Western Pacific regions were comparatively underrepresented relative to their estimated share of the population. This distribution reflects the inherent limitations of non-probability convenience sampling and further restricts the generalizability of these findings to the broader UAE population. However, to mitigate this, the survey was distributed across diverse communities and public settings. Additionally, while the achieved sample size closely approximated the calculated target, the study was not specifically powered for subgroup analyses, and such stratified findings should be interpreted as exploratory rather than confirmatory. The self-administered questionnaire, although reviewed for content validity by three experts, did not undergo formal psychometric validation, which may have affected measurement precision.

The study outcome was based on self-reported prior clinically diagnosed varicose veins instead of clinical examination or Doppler ultrasound confirmation, potentially resulting in misclassification bias. Clinically standardized images of varicose veins were used to improve the accuracy of self-reported cases and awareness among the population. However, it does not replace clinical examination and diagnosis. This approach was utilized to provide objective and performance-based awareness, minimizing recall bias and subjective interpretation. Furthermore, self-reported data on lifestyle and medical factors assessed among the study population may introduce a recall bias.

### 4.5 Conclusion

The prevalence of self-reported varicose veins with prior clinical diagnosis among this convenience sample of adult residents in the United Arab Emirates was 15.05%; as a non-probability sample, this estimate should not be interpreted as representative of the general UAE population. Nonmodifiable factors, including advancing age, and genetic predisposition, alongside modifiable factors like obesity, prolonged standing, and self-reported low hydration and magnesium levels, were significantly associated with higher odds after adjustment. These findings are hypothesis generating and support the need for targeted public health interventions to address venous health and reinforce the clinical importance of varicose veins as a treatable condition, not just a cosmetic concern. To the best of our knowledge, this study provides the first preliminary sample-based prevalence estimate of self-reported varicose veins with prior medical diagnosis among the residing population in the UAE. However, due to the non-probability sampling and self-reported diagnostic, lifestyle, and medical data, the findings should be interpreted as hypothesis-generating and should be followed by clinically validated population-based studies.

## Data Availability

The raw data supporting the conclusions of this article will be made available by the authors, without undue reservation.
