# Supplementary material for: A survey of self-reported varicose veins with prior clinical diagnosis among adults residing in the United Arab Emirates: a cross-sectional study examining associated factors
Source: Front Epidemiol. 2026 Sep 11;6:1916966. doi: 10.3389/fepid.2026.1916966 (PMC13612532; doi:10.3389/fepid.2026.1916966)
Supplement: Supplementary file 1 [file Datasheet1.pdf]

frontiers

Supplementary Material

1 Supplementary Table 1: Association between sociodemographic variables and prevalence of varicose veins with prior clinical diagnosis

Table S1: Association between sociodemographic variables (N = 1043)

Variables		Prevalence		p-value
		No	Yes	
		No. (%)	No. (%)	
Age	less than 30 years	277 (95.8)	12 (4.2)	<.001
	between 30 and 45 years	399 (84.4)	74 (15.6)	
	above 45 years	210 (74.7)	71(25.3)	
Gender	Male	427(88)	58(12)	.009
	Female	459(82.3)	99(17.7)	
Nationality	EMR – Eastern Mediterranean Region	122 (69.3)	54(30.7)	<.001
	SEAR—South-East Asia Region	636 (89.1)	78(10.9)	
	WPR - Western Pacific Region	78 (89.7)	9(10.3)	
	Others	50(75.8)	16(24.2)	
BMI	Normal weight	412 (92)	36(8)	<.001
	Overweight	371 (83.9)	71(16.1)	
	Obese	103 (67.3)	50(32.7)	
Employment status	Not employed	222 (85.7)	37(14.3)	.691

	Employed	664 (84.7)	120(15.3)	

VV = Varicose Veins, BMI = Body Mass Index, Others = African Region (Nigeria, Kenya, Ethiopia, Sudan); European Region (UK, Germany, France); Region of the Americas (USA, Canada, Brazil). The association of sociodemographic characteristics with the prevalence of varicose veins was determined using Pearson's chi-square tests. p-values indicate statistical significance (p<0.05).

2 Supplementary Table 2: Association between lifestyle variables and prevalence of varicose veins with prior clinical diagnosis

Table S2: Association between lifestyle variables (N = 1043)

Variables		Prevalence		p-value
		No	Yes	
		No. (%)	No. (%)	
Exercise routine	No	227(79.4)	59(20.6)	.002
	Yes	659(87.1)	98(12.9)	
Smoking status	No	649(85.8)	107(14.2)	.187
	Yes	237(82.6)	50(17.4)	
Alcohol consumption	No	669 (84.7)	121(15.3)	.674
	Yes	217 (85.8)	36(14.2)	
Hydration status	Less than 2 liters	447(80)	112(20)	<.001
	More than 3 liters	439(90.7)	45(9.3)	
Caffeine consumption	Low or None (< 100 mg)	342(90.2)	37(9.8)	<.001
	Moderate to high (100mg to 400mg or more)	544(81.9)	120(18.1)	
Hours spent sitting daily	Less than 2 hours	68 (74.7)	23(25.3)	<.001
	2 to 4 hours	219 (80.8)	52(19.2)	
	4 to 6 hours	347 (89.2)	42(10.8)	
	More than 6 hours	252(86.3)	40(13.7)	
Hours spent standing daily	Less than 2 hours	197(88.7)	25(11.3)	<.001
	2 to 4 hours	291(93.6)	20(6.4)	
	4 to 6 hours	236(86.4)	37(13.6)	
	More than 6 hours	162(68.4)	75(31.6)	

VV = Varicose Veins. The association of lifestyle factors with the prevalence of varicose veins was determined using Pearson's chi-square tests. p-values indicate statistical significance (p<0.05).

3 Supplementary Table 3: Association between medical variables and prevalence of varicose veins with prior clinical diagnosis

Table S3: Association between medical factors (N = 1043)

**Variables**		**Prevalence**		**p Value**
		**No (%)**	**Yes**	
Diabetes	No	791 (87.4)	114 (12.6)	<.001
	Yes	95(68.8)	43(31.2)	
Hypertension	No	731 (88.4)	96(11.6)	<.001
	Yes	155 (71.8)	61(28.2)	
High cholesterol	No	745 (87.2)	109(12.8)	<.001
	Yes	141 (74.6)	48(25.4)	
Arthritis	No	855 (86.5)	133(13.5)	<.001
	Yes	31 (56.4)	24(43.6)	
Hormonal disorders (PCOS, Thyroid)	No	793 (86.8)	121(13.2)	<.001
	Yes	93 (72.1)	36(27.9)	
COPD, Bronchitis, Asthma, emphysema, and breathing problems	No	831 (85.8)	138(14.2)	.008
	Yes	55 (74.3)	19(25.7)	
Anemia	No	738 (86.5)	115(13.5)	.003
	Yes	148(77.9)	42(22.1)	
Low magnesium	No	822 (86.9)	124(13.1)	<.001
	Yes	64(66)	33(34)	
Constipation or strain during defecation	No	706 (88.4)	93(11.6)	<.001
	Yes	180(73.8)	64(26.2)	
Family history of varicose veins	No	523 (95.1)	27(4.9)	<.001
	Yes.	145 (57.8)	106(42.2)	
	I do not know	218 (90.1)	24(9.9)	

PCOS=polycystic ovarian syndrome; COPD=Chronic Obstructive Pulmonary Disease; VV=Varicose Veins. The association of medical factors with the prevalence of varicose veins was determined using Pearson's chi-square tests. p-values indicate statistical significance ($p<0.05$).
